# Volume measures for linkage disequilibrium

**DOI:** 10.1186/1471-2156-7-54

**Published:** 2006-11-17

**Authors:** Yuguo Chen, Chia-Ho Lin, Chiara Sabatti

**Affiliations:** 1 Department of Statistics, University of Illinois at Urbana-Champaign, Champaign IL 61820, USA; 2 Department of Statistics, UCLA, Los Angeles CA 90095-1554, USA; 3 Department of Human Genetics, UCLA, Los Angeles CA 90095-7088, USA

## Abstract

**Background:**

Defining measures of linkage disequilibrium (LD) that have good small sample properties and are applicable to multiallelic markers poses some challenges. The potential of volume measures in this context has been noted before, but their use has been hampered by computational challenges.

**Results:**

We design a sequential importance sampling algorithm to evaluate volume measures on *I *× *J *tables. The algorithm is implemented in a C routine as a complement to exhaustive enumeration. We make the C code available as open source. We achieve fast and accurate evaluation of volume measures in two dimensional tables.

**Conclusion:**

Applying our code to simulated and real datasets reinforces the belief that volume measures are a very useful tool for LD evaluation: they are not inflated in small samples, their definition encompasses multiallelic markers, and they can be computed with appreciable speed.

## Background

Linkage disequilibrium (LD) is the term used in genetics to indicate association between the qualitative random variables corresponding to alleles at different polymorphic sites. Measuring the levels of linkage disequilibrium is important for gene mapping and increasing our understanding of genome architecture. The current literature documents agreement only on the definition of measures of LD for biallelic markers. Consider two markers, with alleles *A*, *a *and *B*, *b*. Their haplotype distribution can be synthetically described as:

π=BbAxp−xpaq−x1−p−q+x1−pq1−q1.     (1)
 MathType@MTEF@5@5@+=feaafiart1ev1aaatCvAUfKttLearuWrP9MDH5MBPbIqV92AaeXatLxBI9gBaebbnrfifHhDYfgasaacH8akY=wiFfYdH8Gipec8Eeeu0xXdbba9frFj0=OqFfea0dXdd9vqai=hGuQ8kuc9pgc9s8qqaq=dirpe0xb9q8qiLsFr0=vr0=vr0dc8meaabaqaciaacaGaaeqabaqabeGadaaakeaaiiGacqWFapaCcqGH9aqpfaqabeabeqfaubqaaaqaaiabdkeacbqaaiabdkgaIbqaaaqaaiabdgeabbqaaiabdIha4bqaaiabdchaWjabgkHiTiabdIha4bqaaiabdchaWbqaaiabdggaHbqaaiabdghaXjabgkHiTiabdIha4bqaaiabigdaXiabgkHiTiabdchaWjabgkHiTiabdghaXjabgUcaRiabdIha4bqaaiabigdaXiabgkHiTiabdchaWbqaaaqaaiabdghaXbqaaiabigdaXiabgkHiTiabdghaXbqaaiabigdaXaaacqGGUaGlcaWLjaGaaCzcamaabmaabaGaeGymaedacaGLOaGaayzkaaaaaa@54F3@

Fixing the marginals *p *and *q*, the distribution *π *is completely identified by the probability *x *of the haplotype (*A*, *B*). The discrepancy of a generic *π *from the distribution under linkage equilibrium, can be quantified simply by *D *= (*x - pq*). Measures of LD are defined as the standardized values of *D*. Two common such measures are R2=(x−pq)2pq(1−p)(1−q)
 MathType@MTEF@5@5@+=feaafiart1ev1aaatCvAUfKttLearuWrP9MDH5MBPbIqV92AaeXatLxBI9gBaebbnrfifHhDYfgasaacH8akY=wiFfYdH8Gipec8Eeeu0xXdbba9frFj0=OqFfea0dXdd9vqai=hGuQ8kuc9pgc9s8qqaq=dirpe0xb9q8qiLsFr0=vr0=vr0dc8meaabaqaciaacaGaaeqabaqabeGadaaakeaacqWGsbGudaahaaWcbeqaaiabikdaYaaakiabg2da9maalaaabaGaeiikaGIaemiEaGNaeyOeI0IaemiCaaNaemyCaeNaeiykaKYaaWbaaSqabeaacqaIYaGmaaaakeaacqWGWbaCcqWGXbqCcqGGOaakcqaIXaqmcqGHsislcqWGWbaCcqGGPaqkcqGGOaakcqaIXaqmcqGHsislcqWGXbqCcqGGPaqkaaaaaa@44F3@ and D′=(x−pq)Dmax
 MathType@MTEF@5@5@+=feaafiart1ev1aaatCvAUfKttLearuWrP9MDH5MBPbIqV92AaeXatLxBI9gBaebbnrfifHhDYfgasaacH8akY=wiFfYdH8Gipec8Eeeu0xXdbba9frFj0=OqFfea0dXdd9vqai=hGuQ8kuc9pgc9s8qqaq=dirpe0xb9q8qiLsFr0=vr0=vr0dc8meaabaqaciaacaGaaeqabaqabeGadaaakeaacuWGebargaqbaiabg2da9maalaaabaGaeiikaGIaemiEaGNaeyOeI0IaemiCaaNaemyCaeNaeiykaKcabaGaemiraqecbiGae8xBa0Mae8xyaeMae8hEaGhaaaaa@3B02@, where *Dmax *is min(*p*(1 - *q*), *q*(1 - *p*)) when the numerator is positive, and min(*pq*, (1 - *p*) (1 - *q*)) otherwise. The definition of *R*^2 ^can be understood by considering the alleles as realizations of quantitative random variables (with values 0 and 1), among which we calculate a correlation coefficient. The measure *R*^2 ^ranges between 0 and 1, and it is equal to 1 only when two entries of the table in (1) are equal to 0. The measure *D' *ranges, by definition, between -1 and 1, and its absolute value is equal to 1 whenever one entry of the table in (1) is equal to 0. There is a large literature discussing the choice of these measures (see, for example, [1]). Typically, *R*^2 ^is preferred when the focus is on the predictability of one polymorphism given the other (and hence it is often used in power studies for association designs). *D'*, instead, is the measure of choice to assess recombination patterns (haplotypes blocks have often been defined on the basis of *D'*). Despite their effectiveness, these measures suffer from two limitations: (a) they are not easily generalizable to multiallelic markers; (b) they are defined on the population haplotype distribution, and their performance can be rather unsatisfactory when applied to the empirical distribution derived from a finite sample.

With regard to point (a), it is clear that the definitions of *R*^2 ^and *D' *are based on properties of the joint distribution of two biallelic markers and their generalization is not immediate. A partial solution is to evaluate *R*^2 ^or *D' *on all 2 × 2 sub-tables obtainable from the joint distribution of two multiallelic markers and then summarize these results in one value. However, this measure is not easily interpretable and does not have good small sample properties (see the discussion in the following paragraphs).

Finally, let us remark how the problem of defining measures of disequilibrium generalizable to *I *× *J *tables remains actual, even if the current high density genotyping efforts are focused on biallelic markers as SNPs. Often we are interested in studying the relation between SNPs haplotypes at different loci: these can be considered as qualitative variables with multiple levels, just as multiallelic markers.

With regard to point (b), *R*^2 ^and *D' *are defined and studied assuming that the population haplotype distribution is known [2]. In practice, this is rarely (if ever) the case: the sample haplotype frequencies provide an estimate of the population frequencies, and these estimates are used, following the plug-in principle, to obtain estimates of *R*^2 ^and *D'*. This approach encounters some difficulties in the case of *D'*. If a SNP has a low minor allele frequency, it is quite possible that the rare haplotype that carries it, is not observed in a small sample. This leads to a *D' *being equal to 1, irrespective of the level of linkage disequilibrium. Detailed analysis of this phenomena is available in [3] and [4]. To avoid spurious results, researchers often calculate empirical confidence intervals for *D' *using resampling schemes (see, for example, [5]). While this certainly takes care of the variability of *D'*, it does not result in a "correct" measure of linkage disequilibrium and it clearly comes with a substantial computational cost. Moreover, the fact that the values of *D' *are inflated in the presence of rare alleles makes it difficult to obtain a multiallelic version of *D' *based on pooling statistics: as mentioned above, it is quite likely for a particular haplotype of multiallelic markers to have very low population frequency, resulting in zero observed counts. Volume measures [6-8] both take effectively into account the variability due to sample size and are immediately applicable to multiallelic markers. Let us first recall the main idea of volume measures. For distributions like (1), volume measures can be described as a different strategy for normalizing *D *with reference to the class C
 MathType@MTEF@5@5@+=feaafiart1ev1aaatCvAUfKttLearuWrP9MDH5MBPbIqV92AaeXatLxBI9gBamrtHrhAL1wy0L2yHvtyaeHbnfgDOvwBHrxAJfwnaebbnrfifHhDYfgasaacH8akY=wiFfYdH8Gipec8Eeeu0xXdbba9frFj0=OqFfea0dXdd9vqai=hGuQ8kuc9pgc9s8qqaq=dirpe0xb9q8qiLsFr0=vr0=vr0dc8meaabaqaciaacaGaaeqabaWaaeGaeaaakeaaimaacqWFce=qaaa@3825@ of distributions with the same marginals *p *and *q*. Rather than dividing the observed *D *by its maximum value over distributions in C
 MathType@MTEF@5@5@+=feaafiart1ev1aaatCvAUfKttLearuWrP9MDH5MBPbIqV92AaeXatLxBI9gBamrtHrhAL1wy0L2yHvtyaeHbnfgDOvwBHrxAJfwnaebbnrfifHhDYfgasaacH8akY=wiFfYdH8Gipec8Eeeu0xXdbba9frFj0=OqFfea0dXdd9vqai=hGuQ8kuc9pgc9s8qqaq=dirpe0xb9q8qiLsFr0=vr0=vr0dc8meaabaqaciaacaGaaeqabaWaaeGaeaaakeaaimaacqWFce=qaaa@3825@, one evaluates the proportion of distributions in C
 MathType@MTEF@5@5@+=feaafiart1ev1aaatCvAUfKttLearuWrP9MDH5MBPbIqV92AaeXatLxBI9gBamrtHrhAL1wy0L2yHvtyaeHbnfgDOvwBHrxAJfwnaebbnrfifHhDYfgasaacH8akY=wiFfYdH8Gipec8Eeeu0xXdbba9frFj0=OqFfea0dXdd9vqai=hGuQ8kuc9pgc9s8qqaq=dirpe0xb9q8qiLsFr0=vr0=vr0dc8meaabaqaciaacaGaaeqabaWaaeGaeaaakeaaimaacqWFce=qaaa@3825@ that have a smaller difference from the distribution under independence than *D*. In general, we can consider any quantification of the discrepancy between a generic distribution *π *and the distribution under independence. When *π *is known, a volume measure is defined as the probability that a distribution selected uniformly among all possible ones with the same margins as *π *results in a lower discrepancy from equilibrium. If, however, the population haplotype distribution *π *is unknown, and a sample of size *n *is available, volume measures are defined directly on the contingency table summarizing the data, avoiding spurious effects due to the sample size. To clarify this point, let us consider again the case of two biallelic markers. If the population distribution (1) is known, we define *Dvol*, the volume measure equivalent to *D'*, as the ratio of two volumes *V*_1_/*V*_2_. *V*_1 _is the volume of the space of all distributions with marginals equal to *p *and *q*, and *Pr*(0, 0) = *z *such that (*x - pq*) (*z - pq*) ≥ 0 and |*x - pq*| > |*z - pq*|. *V*_2 _is the volume of the space of distributions that satisfy all but the last one of the constraints for *V*_1_. A simple geometric argument shows that *Dvol = D'*. (See Additional file 1 for a graphical illustration). Suppose now, instead, that we do not know the population distribution (1), but we obtain a sample of size *n *from it, leading to the contingency table *F*

F=BbAf11f12r1af21f22r2c1c2n,
 MathType@MTEF@5@5@+=feaafiart1ev1aaatCvAUfKttLearuWrP9MDH5MBPbIqV92AaeXatLxBI9gBaebbnrfifHhDYfgasaacH8akY=wiFfYdH8Gipec8Eeeu0xXdbba9frFj0=OqFfea0dXdd9vqai=hGuQ8kuc9pgc9s8qqaq=dirpe0xb9q8qiLsFr0=vr0=vr0dc8meaabaqaciaacaGaaeqabaqabeGadaaakeaacqWGgbGrcqGH9aqpfaqabeabeqfaubqaaaqaaiabdkeacbqaaiabdkgaIbqaaaqaaiabdgeabbqaaiabdAgaMnaaBaaaleaacqaIXaqmcqaIXaqmaeqaaaGcbaGaemOzay2aaSbaaSqaaiabigdaXiabikdaYaqabaaakeaacqWGYbGCdaWgaaWcbaGaeGymaedabeaaaOqaaiabdggaHbqaaiabdAgaMnaaBaaaleaacqaIYaGmcqaIXaqmaeqaaaGcbaGaemOzay2aaSbaaSqaaiabikdaYiabikdaYaqabaaakeaacqWGYbGCdaWgaaWcbaGaeGOmaidabeaaaOqaaaqaaiabdogaJnaaBaaaleaacqaIXaqmaeqaaaGcbaGaem4yam2aaSbaaSqaaiabikdaYaqabaaakeaacqWGUbGBaaGaeiilaWcaaa@4E4C@

with row sums equal to *r*_1_, *r*_2 _and column sums *c*_1_, *c*_2_. Consider now the set Ω of tables *T*, with row and column sums *r *= (*r*_1_, *r*_2_) and *c *= (*c*_1_, *c*_2_). We define *Dvol*(*F*) as the fraction of contingency tables *T *in Ω that lead to a value |*t*_11 _- *c*_1_*r*_1_/*n*| smaller than |*f*_11 _- *c*_1_*r*_1_/*n*| among those for which (*t*_11 _- *r*_1_*c*_1_/*n*) (*f*_11 _- *r*_1_*c*_1_/*n*) > 0.

This *Dvol *value will be different from *D'*, calculated by treating *f*_*ij*_/*n *as population frequencies. For example, the fact that one table entry is equal to zero will not be sufficient to guarantee *Dvol *= 1. To make this point clearer, however, it is appropriate to consider a more general and precise definition. In the remainder of the paper, we will only concern ourselves with volume measures defined on haplotype sample frequencies.

## Implementation

Consider the table of observed haplotypes counts *F*:

F=B1B2⋯BJA1f11f12⋯f1Jr1⋮⋮⋮⋱⋮⋮AIfI1fI2⋯fIJrIc1c2⋯cJn
 MathType@MTEF@5@5@+=feaafiart1ev1aaatCvAUfKttLearuWrP9MDH5MBPbIqV92AaeXatLxBI9gBaebbnrfifHhDYfgasaacH8akY=wiFfYdH8Gipec8Eeeu0xXdbba9frFj0=OqFfea0dXdd9vqai=hGuQ8kuc9pgc9s8qqaq=dirpe0xb9q8qiLsFr0=vr0=vr0dc8meaabaqaciaacaGaaeqabaqabeGadaaakeaacqWGgbGrcqGH9aqpfaqabeqbgqvauvqaaaqaaiabdkeacnaaBaaaleaacqaIXaqmaeqaaaGcbaGaemOqai0aaSbaaSqaaiabikdaYaqabaaakeaacqWIVlctaeaacqWGcbGqdaWgaaWcbaGaemOsaOeabeaaaOqaaaqaaiabdgeabnaaBaaaleaacqaIXaqmaeqaaaGcbaGaemOzay2aaSbaaSqaaiabigdaXiabigdaXaqabaaakeaacqWGMbGzdaWgaaWcbaGaeGymaeJaeGOmaidabeaaaOqaaiabl+UimbqaaiabdAgaMnaaBaaaleaacqaIXaqmcqWGkbGsaeqaaaGcbaGaemOCai3aaSbaaSqaaiabigdaXaqabaaakeaacqWIUlstaeaacqWIUlstaeaacqWIUlstaeaacqWIXlYtaeaacqWIUlstaeaacqWIUlstaeaacqWGbbqqdaWgaaWcbaGaemysaKeabeaaaOqaaiabdAgaMnaaBaaaleaacqWGjbqscqaIXaqmaeqaaaGcbaGaemOzay2aaSbaaSqaaiabdMeajjabikdaYaqabaaakeaacqWIVlctaeaacqWGMbGzdaWgaaWcbaGaemysaKKaemOsaOeabeaaaOqaaiabdkhaYnaaBaaaleaacqWGjbqsaeqaaaGcbaaabaGaem4yam2aaSbaaSqaaiabigdaXaqabaaakeaacqWGJbWydaWgaaWcbaGaeGOmaidabeaaaOqaaiabl+UimbqaaiabdogaJnaaBaaaleaacqWGkbGsaeqaaaGcbaGaemOBa4gaaaaa@71F1@

where *B*_*i *_represent alleles at marker B and *A*_*i *_alleles at marker A. Let Ω be the set of all tables *T *with row and column sums equal to *r*_1_, ..., *r*_*I *_and *c*_1_, ..., *c*_*J*_, respectively. Given a criterion to quantify the discrepancy between *F *and the table expected under independence (linkage equilibrium), a volume measure is defined as the proportion of tables *T *∈ Ω that lead to a smaller discrepancy value. If the recorded discrepancy is the biggest possible, then the volume measure will have value close to 1 (the exact value 1 will be attained as the sample size increases to ∞). Conversely, if all other tables lead to larger discrepancies, the volume measure will be zero.

One may notice that this definition of volume measure is similar to one minus the *p*-value for a test of independence. Indeed, volume measures are related to the "volume test," an original notion introduced by Hotelling [8], and the effect of sample size on the measures is very much the same as its effect on a *p*-value. The key difference between volume measures and variants of the commonly used Fisher's exact test for independence is that in the case of volume measures, the relevant proportion of tables is evaluated assuming that all tables with the same margins are equally probable, while in the case of Fisher's exact test tables are generated under the hypothesis of independence. Because of this, volume measures and Fisher's exact tests answer two very different questions: the first compares the observed table to all tables with the same margins, while the second one assesses the likelihood of the observed table under independence. A thorough discussion of the different interpretations and uses of these two approaches can be found in [7]. In order to concretely evaluate volume measures, one has to choose a criterion for discrepancy and be able to explore the space of tables with fixed margins to evaluate the required proportions. We start illustrating the first point by focusing on three specific measures: a) *Dvol*, which is defined only on 2 × 2 tables and coincides with *D' *when the population haplotype distribution is known; b) *Mvol*, which is a generalization of *Dvol *to multiallelic markers; c) *Hvol*, which is based on expected homozygosity and captures information that is close to the one described by *R*^2^, although it can be defined on tables with any number of entries.

When *I *= *J *= 2, let Ω_1 _= {*T *: *t*_*i*+ _= *r*_*i*_, *t*_+*j *_= *c*_*j*_, (*t*_11 _- *r*_1_*c*_1_/*n*)(*f*_11 _- *r*_1_*c*_1_/*n*) > 0}. We then define *Dvol *as

Dvol(F)=1|Ω1|∑T∈Ω11{M(T)<M(F)},
 MathType@MTEF@5@5@+=feaafiart1ev1aaatCvAUfKttLearuWrP9MDH5MBPbIqV92AaeXatLxBI9gBaebbnrfifHhDYfgasaacH8akY=wiFfYdH8Gipec8Eeeu0xXdbba9frFj0=OqFfea0dXdd9vqai=hGuQ8kuc9pgc9s8qqaq=dirpe0xb9q8qiLsFr0=vr0=vr0dc8meaabaqaciaacaGaaeqabaqabeGadaaakeaacqWGebarcqWG2bGDcqWGVbWBcqWGSbaBcqGGOaakcqWGgbGrcqGGPaqkcqGH9aqpdaWcaaqaaiabigdaXaqaaiabcYha8jabfM6axnaaBaaaleaacqaIXaqmaeqaaOGaeiiFaWhaamaaqafabaGaeGymaeZaaSbaaSqaaiabcUha7jabd2eanjabcIcaOiabdsfaujabcMcaPiabgYda8iabd2eanjabcIcaOiabdAeagjabcMcaPiabc2ha9bqabaaabaGaemivaqLaeyicI4SaeuyQdC1aaSbaaWqaaiabigdaXaqabaaaleqaniabggHiLdGccqGGSaalaaa@51F8@

where M(T)=∑i,j(tij−ricj/n)2ricj/n
 MathType@MTEF@5@5@+=feaafiart1ev1aaatCvAUfKttLearuWrP9MDH5MBPbIqV92AaeXatLxBI9gBaebbnrfifHhDYfgasaacH8akY=wiFfYdH8Gipec8Eeeu0xXdbba9frFj0=OqFfea0dXdd9vqai=hGuQ8kuc9pgc9s8qqaq=dirpe0xb9q8qiLsFr0=vr0=vr0dc8meaabaqaciaacaGaaeqabaqabeGadaaakeaacqWGnbqtcqGGOaakcqWGubavcqGGPaqkcqGH9aqpdaaeqaqaamaalaaabaGaeiikaGIaemiDaq3aaSbaaSqaaiabdMgaPjabdQgaQbqabaGccqGHsislcqWGYbGCdaWgaaWcbaGaemyAaKgabeaakiabdogaJnaaBaaaleaacqWGQbGAaeqaaOGaei4la8IaemOBa4MaeiykaKYaaWbaaSqabeaacqaIYaGmaaaakeaacqWGYbGCdaWgaaWcbaGaemyAaKgabeaakiabdogaJnaaBaaaleaacqWGQbGAaeqaaOGaei4la8IaemOBa4gaaaWcbaGaemyAaKMaeiilaWIaemOAaOgabeqdcqGHris5aaaa@4FC0@.

For general *I *× *J *tables, recall that Ω denotes the set of all contingency tables with the same row and column sums as *F*: Ω = {*T *: *t*_*i*+ _= *r*_*i*_, *t*_+*j *_= *c*_*j*_}. Then, we define

Mvol(F)=1|Ω|∑T∈Ω1{M(T)<M(F)}.
 MathType@MTEF@5@5@+=feaafiart1ev1aaatCvAUfKttLearuWrP9MDH5MBPbIqV92AaeXatLxBI9gBaebbnrfifHhDYfgasaacH8akY=wiFfYdH8Gipec8Eeeu0xXdbba9frFj0=OqFfea0dXdd9vqai=hGuQ8kuc9pgc9s8qqaq=dirpe0xb9q8qiLsFr0=vr0=vr0dc8meaabaqaciaacaGaaeqabaqabeGadaaakeaacqWGnbqtcqWG2bGDcqWGVbWBcqWGSbaBcqGGOaakcqWGgbGrcqGGPaqkcqGH9aqpdaWcaaqaaiabigdaXaqaaiabcYha8jabfM6axjabcYha8baadaaeqbqaaiabigdaXmaaBaaaleaacqGG7bWEcqWGnbqtcqGGOaakcqWGubavcqGGPaqkcqGH8aapcqWGnbqtcqGGOaakcqWGgbGrcqGGPaqkcqGG9bqFaeqaaaqaaiabdsfaujabgIGiolabfM6axbqab0GaeyyeIuoakiabc6caUaaa@4FC0@

The definition above should clarify how *Mvol *is closely related to *Dvol*, and the difference between the two is that *Mvol *does not consider the "sign" of the association, a notion that is undefined in generic *I *× *J *tables.

Letting H(T)=∑i,jtij2−∑iri2∑jcj2/n2
 MathType@MTEF@5@5@+=feaafiart1ev1aaatCvAUfKttLearuWrP9MDH5MBPbIqV92AaeXatLxBI9gBaebbnrfifHhDYfgasaacH8akY=wiFfYdH8Gipec8Eeeu0xXdbba9frFj0=OqFfea0dXdd9vqai=hGuQ8kuc9pgc9s8qqaq=dirpe0xb9q8qiLsFr0=vr0=vr0dc8meaabaqaciaacaGaaeqabaqabeGadaaakeaacqWGibascqGGOaakcqWGubavcqGGPaqkcqGH9aqpdaaeqaqaaiabdsha0naaDaaaleaacqWGPbqAcqWGQbGAaeaacqaIYaGmaaaabaGaemyAaKMaeiilaWIaemOAaOgabeqdcqGHris5aOGaeyOeI0YaaabeaeaacqWGYbGCdaqhaaWcbaGaemyAaKgabaGaeGOmaidaaaqaaiabdMgaPbqab0GaeyyeIuoakmaaqababaGaem4yam2aa0baaSqaaiabdQgaQbqaaiabikdaYaaaaeaacqWGQbGAaeqaniabggHiLdGccqGGVaWlcqWGUbGBdaahaaWcbeqaaiabikdaYaaaaaa@4EF5@, we can define the measure *Hvol*:

Hvol(F)=sign(H(F))∑T∈Ω1{|H(T)|<|H(F)|}1{H(T)H(F)≥0}∑T∈Ω1{H(T)H(F)≥0}.
 MathType@MTEF@5@5@+=feaafiart1ev1aaatCvAUfKttLearuWrP9MDH5MBPbIqV92AaeXatLxBI9gBaebbnrfifHhDYfgasaacH8akY=wiFfYdH8Gipec8Eeeu0xXdbba9frFj0=OqFfea0dXdd9vqai=hGuQ8kuc9pgc9s8qqaq=dirpe0xb9q8qiLsFr0=vr0=vr0dc8meaabaqaciaacaGaaeqabaqabeGadaaakeaacqWGibascqWG2bGDcqWGVbWBcqWGSbaBcqGGOaakcqWGgbGrcqGGPaqkcqGH9aqpcqqGZbWCcqqGPbqAcqqGNbWzcqqGUbGBcqGGOaakcqWGibascqGGOaakcqWGgbGrcqGGPaqkcqGGPaqkdaWcaaqaamaaqababaGaeGymaeZaaSbaaSqaaiabcUha7jabcYha8jabdIeaijabcIcaOiabdsfaujabcMcaPiabcYha8jabgYda8iabcYha8jabdIeaijabcIcaOiabdAeagjabcMcaPiabcYha8jabc2ha9bqabaGccqaIXaqmdaWgaaWcbaGaei4EaSNaemisaGKaeiikaGIaemivaqLaeiykaKIaemisaGKaeiikaGIaemOrayKaeiykaKIaeyyzImRaeGimaaJaeiyFa0habeaaaeaacqWGubavcqGHiiIZcqqHPoWvaeqaniabggHiLdaakeaadaaeqaqaaiabigdaXmaaBaaaleaacqGG7bWEcqWGibascqGGOaakcqWGubavcqGGPaqkcqWGibascqGGOaakcqWGgbGrcqGGPaqkcqGHLjYScqaIWaamcqGG9bqFaeqaaaqaaiabdsfaujabgIGiolabfM6axbqab0GaeyyeIuoaaaGccqGGUaGlaaa@7E7B@

We have mentioned how *Hvol *captures information closely related to that of *R*^2^. A careful discussion of the interpretation of LD measures based on homozygosity can be found in [9]. Here it suffices to recall that joint homozygosity relates to a measure of agreement between the two markers and excess in homozygosity indicates that knowledge of the allele value at one marker increases predictive accuracy of the allele values at the other marker. The results of a recent empirical study conducted using homozygosity-based measures are documented in [10].

Note that all the above definitions use the strict inequality sign. The choice of this over ≤ is irrelevant for large *n*, but it makes a difference in the case of small *n*, where strict inequality allows us to better discriminate against apparent association due to small samples.

To evaluate these measures, we need to explore the space of all tables with the same margins. In the case of *I *= *J *= 2, this can be done by simple enumeration. For multiallelic tables enumeration is impractical. An obvious alternative is to restrict one's attention to a sample of possible tables. However, obtaining a sample of tables according to the uniform distribution among all tables with fixed margin (as opposed to according to the Fisher-Yates distribution) is not easy. It is indeed the computational difficulty associated with volume tests [7] and measures [6] that has substantially hindered their wide-spread application. Previous solutions have been proposed with Markov chain Monte Carlo algorithms in [11], as well as rejection sampling (see [12] for a review). The main contribution of this paper is that we have successfully implemented a sequential importance sampling (SIS) algorithm, originally introduced in [12], to evaluate volume measures accurately and in a timely manner. This implementation makes volume measures applicable to high throughput analysis.

To enumerate all tables in Ω_1 _(*I = J = *2), it is useful to notice that *t*_11 _must satisfy

max(0, *r*_1 _+ *c*_1 _- *n*) ≤ *t*_11 _≤ min(*r*_1_, *c*_1_),     (2)

and after *t*_11 _is chosen, we can fill in other entries of the 2 × 2 table by the marginal sum constraints. Therefore we can enumerate tables in Ω_1 _by assigning all possible integers satisfying (2) to *t*_11_, and keeping those tables such that (*t*_11 _- *r*_1_*c*_1_/*n*) has the same sign as in *F*.

We now consider the SIS procedure for *I *× *J *tables. Let *u*(*T*) be the uniform distribution over all tables in Ω. Then *Mvol*(*F*) can be treated as the expectation of the indicator function 1_{*m*(*T*)<*m*(*F*)} _with respect to *u*(*T*). It is hard to sample directly from *u*(*T*). The idea of importance sampling is to sample tables from another proposal distribution *g*(*T*), and then estimate *Mvol*(*F*) by

∑ℓ=1L1{M(Tℓ)<M(F)}u(Tℓ)g(Tℓ)∑ℓ=1Lu(Tℓ)g(Tℓ)=∑ℓ=1L1{M(Tℓ)<M(F)}1g(Tℓ)∑ℓ=1L1g(Tℓ),
 MathType@MTEF@5@5@+=feaafiart1ev1aaatCvAUfKttLearuWrP9MDH5MBPbIqV92AaeXatLxBI9gBaebbnrfifHhDYfgasaacH8akY=wiFfYdH8Gipec8Eeeu0xXdbba9frFj0=OqFfea0dXdd9vqai=hGuQ8kuc9pgc9s8qqaq=dirpe0xb9q8qiLsFr0=vr0=vr0dc8meaabaqaciaacaGaaeqabaqabeGadaaakeaadaWcaaqaamaaqadabaGaeGymaeZaaSbaaSqaaiabcUha7jabd2eanjabcIcaOiabdsfaunaaBaaameaacqWItecBaeqaaSGaeiykaKIaeyipaWJaemyta0KaeiikaGIaemOrayKaeiykaKIaeiyFa0habeaakmaalaaabaGaemyDauNaeiikaGIaemivaq1aaSbaaSqaaiabloriSbqabaGccqGGPaqkaeaacqWGNbWzcqGGOaakcqWGubavdaWgaaWcbaGaeS4eHWgabeaakiabcMcaPaaaaSqaaiabloriSjabg2da9iabigdaXaqaaiabdYeambqdcqGHris5aaGcbaWaaabmaeaadaWcaaqaaiabdwha1jabcIcaOiabdsfaunaaBaaaleaacqWItecBaeqaaOGaeiykaKcabaGaem4zaCMaeiikaGIaemivaq1aaSbaaSqaaiabloriSbqabaGccqGGPaqkaaaaleaacqWItecBcqGH9aqpcqaIXaqmaeaacqWGmbata0GaeyyeIuoaaaGccqGH9aqpdaWcaaqaamaaqadabaGaeGymaeZaaSbaaSqaaiabcUha7jabd2eanjabcIcaOiabdsfaunaaBaaameaacqWItecBaeqaaSGaeiykaKIaeyipaWJaemyta0KaeiikaGIaemOrayKaeiykaKIaeiyFa0habeaakmaalaaabaGaeGymaedabaGaem4zaCMaeiikaGIaemivaq1aaSbaaSqaaiabloriSbqabaGccqGGPaqkaaaaleaacqWItecBcqGH9aqpcqaIXaqmaeaacqWGmbata0GaeyyeIuoaaOqaamaaqadabaWaaSaaaeaacqaIXaqmaeaacqWGNbWzcqGGOaakcqWGubavdaWgaaWcbaGaeS4eHWgabeaakiabcMcaPaaaaSqaaiabloriSjabg2da9iabigdaXaqaaiabdYeambqdcqGHris5aaaakiabcYcaSaaa@8926@

where *T*_1_, ..., *T*_*L *_are *L *independent and identically distributed (i.i.d.) samples from *g*(*T*). SIS generates a table cell by cell by decomposing the proposal distribution *g*(*T*) as

*g*(*T*)*= g*(*t*_11_)*g*(*t*_21_|*t*_11_) ... *g*(*t*_*IJ*_|*t*_*I*-1_, _*J*_, ..., *t*_11_).

Notice that the support for the first entry *t*_11 _is max(0, *r*_1 _+ *c*_1 _- *n*) ≤ *t*_11 _≤ min(*r*_1_, *c*_1_). We sample an integer uniformly from the above range for in, i.e., *g*(*t*_11_) is the uniform distribution on the support of *t*_11_.

Recursively, suppose we have chosen *t*_*i*1 _= ti1∗
 MathType@MTEF@5@5@+=feaafiart1ev1aaatCvAUfKttLearuWrP9MDH5MBPbIqV92AaeXatLxBI9gBaebbnrfifHhDYfgasaacH8akY=wiFfYdH8Gipec8Eeeu0xXdbba9frFj0=OqFfea0dXdd9vqai=hGuQ8kuc9pgc9s8qqaq=dirpe0xb9q8qiLsFr0=vr0=vr0dc8meaabaqaciaacaGaaeqabaqabeGadaaakeaacqWG0baDdaqhaaWcbaGaemyAaKMaeGymaedabaGaey4fIOcaaaaa@3184@ for *i *= 1, ..., *k *- 1. Then the support for *t*_*k*1 _is max⁡(0,(t.1−∑i=1k−1ti1∗)−∑i=k+1Iri)≤tk1≤min⁡(rk,c1−∑i=1k−1ti1∗)
 MathType@MTEF@5@5@+=feaafiart1ev1aaatCvAUfKttLearuWrP9MDH5MBPbIqV92AaeXatLxBI9gBaebbnrfifHhDYfgasaacH8akY=wiFfYdH8Gipec8Eeeu0xXdbba9frFj0=OqFfea0dXdd9vqai=hGuQ8kuc9pgc9s8qqaq=dirpe0xb9q8qiLsFr0=vr0=vr0dc8meaabaqaciaacaGaaeqabaqabeGadaaakeaacyGGTbqBcqGGHbqycqGG4baEdaqadaqaaiabicdaWiabcYcaSiabcIcaOiabdsha0jabc6caUmaaBaaaleaacqaIXaqmaeqaaOGaeyOeI0YaaabmaeaacqWG0baDdaqhaaWcbaGaemyAaKMaeGymaedabaGaey4fIOcaaaqaaiabdMgaPjabg2da9iabigdaXaqaaiabdUgaRjabgkHiTiabigdaXaqdcqGHris5aOGaeiykaKIaeyOeI0YaaabmaeaacqWGYbGCdaWgaaWcbaGaemyAaKgabeaaaeaacqWGPbqAcqGH9aqpcqWGRbWAcqGHRaWkcqaIXaqmaeaacqWGjbqsa0GaeyyeIuoaaOGaayjkaiaawMcaaiabgsMiJkabdsha0naaBaaaleaacqWGRbWAcqaIXaqmaeqaaOGaeyizImQagiyBa0MaeiyAaKMaeiOBa42aaeWaaeaacqWGYbGCdaWgaaWcbaGaem4AaSgabeaakiabcYcaSiabdogaJnaaBaaaleaacqaIXaqmaeqaaOGaeyOeI0YaaabmaeaacqWG0baDdaqhaaWcbaGaemyAaKMaeGymaedabaGaey4fIOcaaaqaaiabdMgaPjabg2da9iabigdaXaqaaiabdUgaRjabgkHiTiabigdaXaqdcqGHris5aaGccaGLOaGaayzkaaaaaa@75ED@. We sample an integer uniformly from the above range for *t*_*k*1_. The procedure is continued until all the entries in the first column have been considered. Then we update the row sums by subtracting the realization of the first column from the original row sum, and sample the second column of the table in the same way.

The computing time and precision of the algorithm are different for 2 × 2 or larger size tables. For 2 × 2 tables, our algorithm simply lists all possible tables with fixed margins. CPU time is then proportional to the total number of tables: usually their enumeration takes a fraction of a second. The algorithm is exact and we do not have approximation errors in the output. For the general case of *I *× *J *tables, the CPU time depends on the number of generated uniform random variables: *I *× *J *× *L *for *L *Monte Carlo samples. It is important to keep in mind that the output of the algorithm is not exact, but an estimate of the true volumes ratio (so different runs will give slightly different results). The precision of the final estimate depends on the number *L *of Monte Carlo samples and how well the proposal distribution in the SIS algorithm approximates the target distribution for a given table. Indeed, the value of the parameter *L *has to be specified by the user. It is advisable to conduct multiple trial runs to estimate the precision of the estimate and select a value of *L *that assures an acceptable precision.

## Results

We now illustrate the performance of our algorithm and the relevance of volume measures with three examples.

### The effect of small sample size on *D' *and *Dvol*

**Figure 1:**
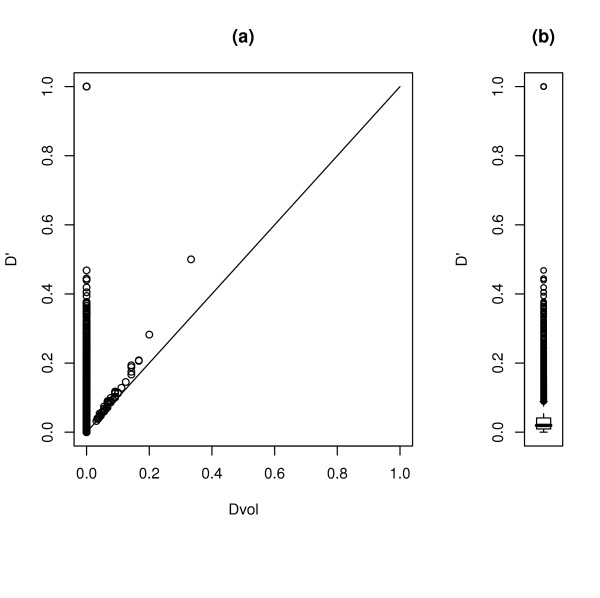
**Comparison of D' and Dvol**. Comparison of *D' *and *Dvol *on tables generated under linkage equilibrium, (a) Scatterplot of the values of *D' *and *Dvol*. (b) Boxplot of the values of *D'*.

It has been noted that *D' *tends to be biased upwards in small samples [3,4]. We conducted a simulation study to illustrate how this problem is less severe when using *Dvol*. We generated 100 two-markers haplotype tables with 200 observations, each under the hypothesis of linkage equilibrium between the markers. The distribution of the frequency of the minor alleles of the simulated SNPs matched a random sample of markers on chromosome 22 that were used in [13]. In a situation where the true population value of *D' *is equal to zero, any sample based estimator is going to be upward biased, since 0 is the minimum value that *D' *can achieve. The point of our investigation was to compare the severity of this bias. Figure 1 illustrates the results: *D' *is always larger than *Dvol*, and it is occasionally equal to 1; *Dvol *is actually equal to zero in the majority of cases.

### Patterns of LD between multiallelic markers

**Figure 2:**
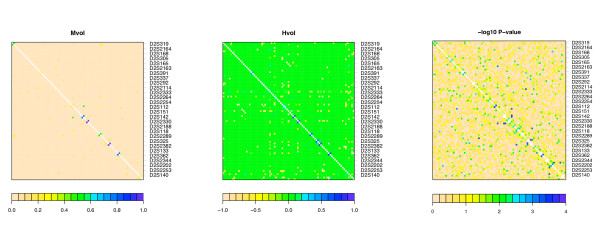
**Measuring LD between multiallelic markers**. Measure of disequilibrium between microsatellites. Each square in this symmetric picture corresponds to a marker pair (the same markers are reported on both rows and columns). The three panels report, from left to right, *Mvol*, *Hvol*, and the negative of the log10 of the *p*-value for a Fisher's exact test of independence.

Our next example focuses on the application of volume measures to multiallelic markers. The data consists of 157 phase-known non-transmitted chromosomes 2 of parents of BP-I persons from the Central Valley of Costa Rica. The chromosomes were typed with 85 markers in the course of the study by [14]. Using volume measures *Mvol *and *Hvol *we were able to evaluate the level of disequilibrium between all the possible marker pairs in this sample. Figure 2 gives a graphical representation of the values of *Mvol *and *Hvol *in this data set as well as the negative of log10 *p*-value for a Fisher exact test of independence. This last one is reported for comparison purposes, as it is often used as a measure of dependence, despite the fact that it is rather inappropriate with this goal [7]. Volume measures make it unnecessary to resort to this unsatisfactory surrogate when comparing multiallelic markers. To analyze these tables, we used *L *= 1, 000, 000 Monte Carlo sample. The average time to evaluate the measures on one table was 48 seconds on a Dell desktop with 2.19 GHz CPU and 384 MB ram.

### Consistency of LD patterns on chr 22 in 12 populations

**Figure 3:**
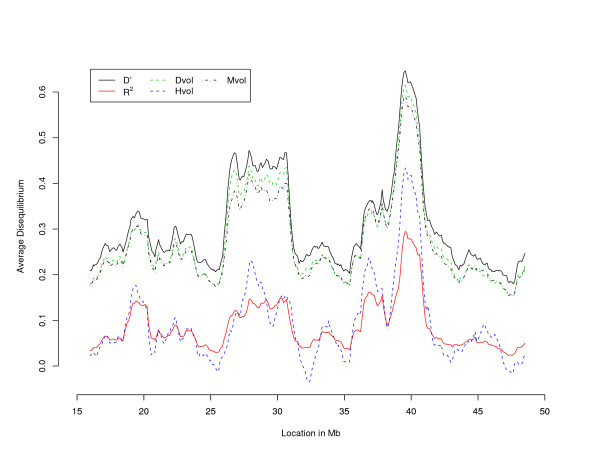
**LD pattern on Chr 22 in a Costa Rican population**. Linkage disequilibrium of chromosome 22 in Costa Rica according to five different measures. *D'*, *R*^2^, *Dvol*, *Mvol *and *Hvol *are represented, respectively, with a solid yellow, a broken green, a solid blue, a broken magenta, and a solid red line. The average value of the measures, between markers that are within a 1.7 Mb window, is plotted against the middle point of the window, with the *x *axis representing the length of chromosome 22.

We have used the measures *D'*, |*R*|, *Dvol*, *Mvol *and *Hvol *to assess the distribution and extent of linkage disequilibrium on chromosome 22 in samples of 200 persons from each of eleven population isolates and in an out-bred Caucasian sample, using 2486 SNP markers spaced at a density of approximately one marker every 13.8 kb. [10,13]. To conduct a complete analysis of the linkage disequilibrium patterns in the 12 population samples, we restricted our attention to the SNPs with sample minor allele frequencies larger than 0.1. We did so for uniformity with previous studies (for example, [15]) and to make sure that our results were not strongly influenced by the rare markers with exceptionally high homozygosity. This leads us to work with 1920 SNPs. Phase was unknown and the two markers haplotypes counts necessary to evaluate pair-wise disequilibrium measures were reconstructed using EM [16]. Five measures, *D'*, *Dvol*, *Mvol*, *R*^2 ^and *Hvol *were calculated for each of the 1,842,240 pairs of SNPs. The results were summarized by averaging the measured disequilibrium within windows of 1.7 Mb sliding along chromosome 22. Figure 3 reports the values of the five measures in the Costa Rica population. The observed relation between the measures is consistent across populations. In particular, it can be noted that the average values of *Dvol *are lower than the ones of *D'*, while clearly exhibiting very similar patterns. This testifies that even if the sample size is moderately large (200 individuals) and only markers with minor allele frequency >0.1 are considered, *D' *is inflated, making a strong case for the use of *Dvol *over *D'*. The values of *Mvol *are very close to the one of *Dvol*, even if *Mvol *are often smaller as expected given the differences in definitions. As far as *Hvol*, one can notice that its values are closer to those of *R*^2 ^than to those of any other measure. Finally, let us observe that the computational time required for evaluating all the volume measures above amounts to an average of 5 minutes for each population on a Dell desktop with 2.19 GHz CPU and 384 MB ram (this is after pairwise haplotypes counts were reconstructed, which required approximately the same amount of time). The substantial difference in computational time with the results reported in the previous subsection is due to the fact that here we are dealing with 2 × 2 tables.

## Conclusion

We describe a novel implementation of a sequential importance sampling algorithm to evaluate volume measures of linkage disequilibrium. We focus on three measures. *Dvol *corresponds conceptually to *D'*, but we show that *Dvol *is not inflated for small sample size. *Mvol *represents a generalization of *Dvol *that can be evaluated on generic *I *× *J *tables. *Hvol *is based on expected homozygosity and measures agreement between markers, so that it captures information similar to that of *R*^2^. However, unlike *R*^2^, *Hvol *can be evaluated on generic *I *× *J *tables.

## Availability and requirements

The source code for evaluating the volume measures described in this paper is available at the following url: .

It is a C program that can be compiled with gcc and requires the libraries math.h, stdlib.h, malloc.h, time.h, and stdio.h.

## Authors' contributions

YC has been responsible mainly for the algorithm and code development. CL has contributed mainly to data analysis. CS has supervised the project.

## Supplementary Material

Additional file 1 **Measures of disequilibrium between biallelic markers**. This .pdf file contains a detailed description of measures of disequilibrium for population haplotype distributions for biallelic markers. We recall the definition of *D' *and *R*^2 ^as well of *Dvol *and *Mvol*. We graphically illustrate the difference in normalization procedure between all of these measures. This makes it easy to see the identity of *D' *and *Dvol *when the population haplotype frequency is known. Click here for file
