# Supplementary material for: Volume measures for linkage disequilibrium
Source: BMC Genet. 2006 Nov 17;7:54. doi: 10.1186/1471-2156-7-54 (PMC1665459; doi:10.1186/1471-2156-7-54)
Supplement: Additional file 1 — Measures of disequilibrium between biallelic markers. This .pdf file contains a detailed description of measures of disequilibrium for population haplotype distributions for biallelic markers. We recall the definition of D' and R2 as well of Dvol and Mvol. We graphically illustrate the difference in normalization procedure between all of these measures. This makes it easy to see the identity of D' and Dvol when the population haplotype frequency is known. [file 1471-2156-7-54-S1.pdf]

Additional file 1
Measures of disequilibrium between biallelic markers

Yuguo Chen[1], Chia-Ho Lin[2], and Chiara Sabatti[2,3]

Department of Statistics, University of Illinois at Urbana-Champaign, Champaign IL 61820[1].

Department of Statistics[2], and Department of Human Genetics[3], UCLA, Los Angeles CA 90095-7088.

October 2006

In this supplementary note, we recall the definition of R^2 and D' on 2×2 contingency tables representing population haplotype frequencies. We then introduce the definition of $Dvol$, $Mvol$ and $Hvol$ in the same context and illustrate the differences between these various measures resorting to simple graphics.

Consider two markers, with alleles A, a and B, b. Their population haplotype distribution can be synthetically described as:

$$\pi = \begin{array}{c|c|c|c} & B & b & \\ \hline A & x & p-x & p \\ \hline a & q-x & 1-p-q+x & 1-p \\ \hline & q & 1-q & 1 \end{array}. \tag{1}$$

Fixing the marginals p and q, the distribution π is completely identified by the probability x of the haplotype (A, B). The $|R|$ measure is derived by assigning the alleles the quantitative values 0,1 and calculating the correlation coefficient between the two random variables corresponding to the two alleles:

$$|R| = \frac{|x - pq|}{\sqrt{pq(1-p)(1-q)}}.$$

It is clear that $|R| \leq 1$, however this bound is quite crude in that the value of 1 can be achieved only when $p = q$ or $p = 1 - q$. That is, $|R|$ is equal to 1 only when knowing the allele at one of the markers always allows perfect prediction of the allele at the other marker (irrespective of which marker is known and which allele value).

The maximum value of D conditional on p and q can also be easily calculated in a 2×2 table. This was done, for example, by Lewontin, who also takes into account the direction of disequilibrium. The resulting D' measure is defined as:

$$D' = \begin{cases} \dfrac{|D|}{\min(p(1-q), q(1-p))} & \text{if} \quad D \geq 0 \\ \\ \dfrac{|D|}{\min(pq, (1-p)(1-q))} & \text{if} \quad D < 0 \end{cases},$$

where we have taken the absolute value of D, since its sign carries no genetically relevant information. Clearly one could define another measure that standardizes D conditional on p and q, irrespective of the sign of D (the denominator would be the maximum of the two different denominators appearing in the D' definition). Note that $D' = 1$ when one of the entries in the contingency table is equal to 0 (while $|R| = 0$ only if two entries are 0): this corresponds to the situation of no recombination having ever occurred between the two markers since the arising of one of the polymorphisms, and hence D' is favored by geneticists that try to measure the amount of recombination in terms of LD.

In the case of 2×2 tables corresponding to known population distributions, it is easy to evaluate volume measures, and it is useful to compare the two measures described above in some detail. In terms of the parameterization (1), the set of values $\max(0, p+q-1) \leq x \leq \min(p, q)$ represents all the possible haplotypes distributions on two biallelic markers with marginal allele frequencies p and q. In Figure 1, the range of x is represented on the horizontal coordinate, a specific value of z corresponding to one distribution is put in evidence, and the values of the curve $|x - pq|$ are drawn. The measures of disequilibrium D' (red) and $|R|$ (green) can be described as ratios of values on the y coordinate: the numerators are indicated with broken lines and the denominators with solid ones. Note that the denominator in $|R|$ typically does not correspond to the achievable maximum for $|x - pq|$. Values of volume measures are, instead, ratios of quantities identifiable on the x axis. Two measures are described in Figure 1: $Dvol$ (red) and $Mvol$ (blue). $Dvol$ is defined as the ratio of the volume of the space of distributions for which $|x - pq| < |z - pq|$ and $(x - pq)(z - pq) \geq 0$ and the volume of the space of distributions for which $(x - pq)(z - pq) \geq 0$. A simple geometric argument shows that in this context (when we are dealing with a known population distribution) $Dvol = D'$. It is important to remark that the equality of D' and $Dvol$ does not hold when we consider the definition of these measures on a finite sample of haplotypes—which is the case we deal with more extensively in the main paper. The $Mvol$ measure is based on Mahalanobis distance between the specific distribution represented by z and the distribution under independence. As in

the 2×2 case, $Mvol$ is equal to $\frac{(x-pq)^2}{pq(1-p)(1-q)}$, the ratio of the blue lines in Figure 1. To further clarify the differences between the considered measures, we illustrated in Figure 2 a case where $p = (1-q)$ and $z = 0$.

Finally, Figure 3 illustrates the definition of $Hvol$ using the same setting as above. Let $H(x)$ be the expected homozygosity associated with the table in (1). Then $Hvol$ for a table z is defined as the ratio of the volume of the space of distributions x for which $|H(x)-H(pq)| < |H(z)-H(pq)|$ and $(H(x)-H(pq))(H(z)-H(pq)) \geq 0$ and the volume of the space of distributions x such that $(H(x)-H(pq))(H(z)-H(pq)) \geq 0$. Note that generally speaking, the parabola defined by $H(x)-H(pq)$ may cross the zero axis two times within the range of possible values for x.

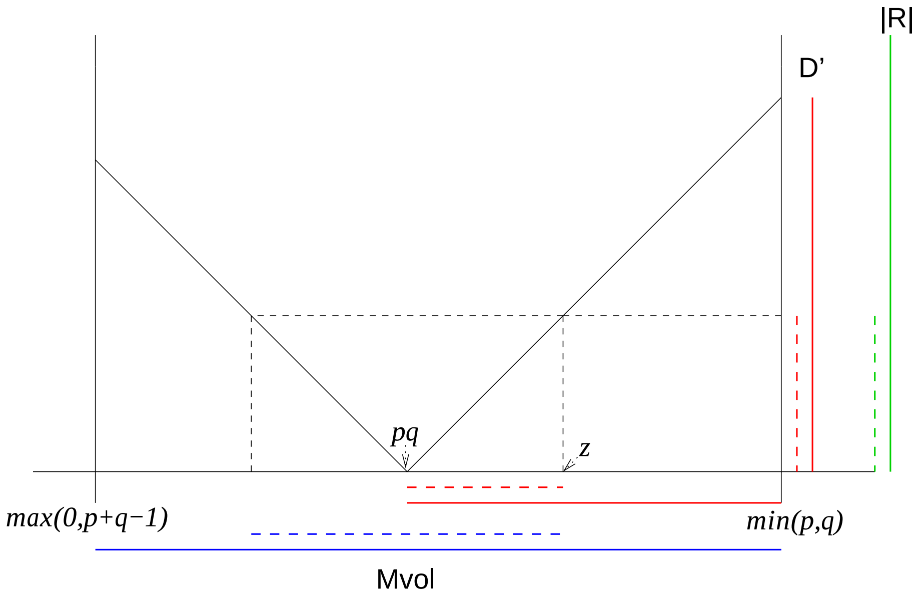

Figure 1: Measures of association on 2×2 tables. The value of the x entry of the table in (1) is displayed on the horizontal coordinate. The point z corresponds to a specific table under consideration. The highlighted point pq corresponds to linkage equilibrium (independence). The values of the measures D', $|R|$, and $Mvol$ for the table identified by z are presented as ratios between broken and solid lines of the following colors respectively: red, green, and blue.

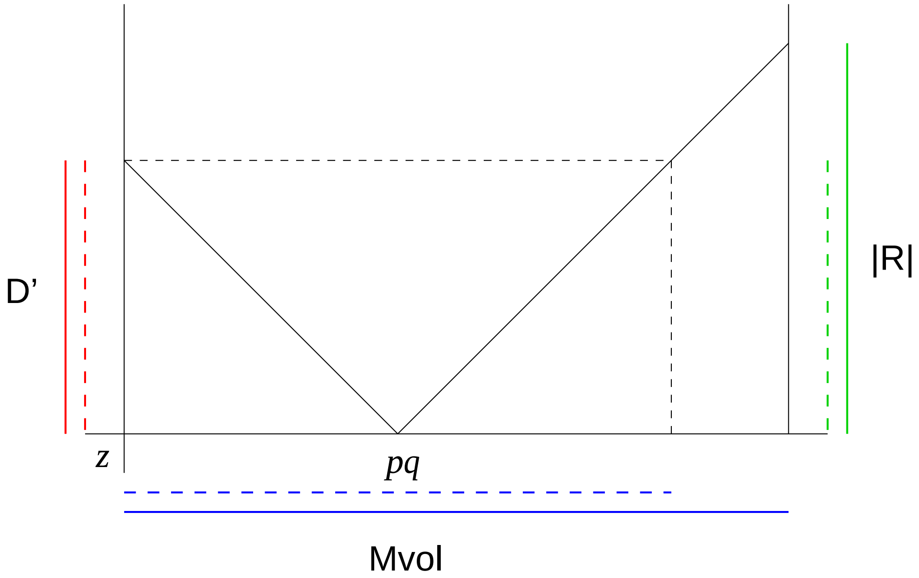

Figure 2: Measures of association on 2×2 tables. The value of the x entry of the table in (1) is displayed on the horizontal coordinate. The point z corresponds to a specific table under consideration. The highlighted point pq corresponds to linkage equilibrium (independence). We assumed here that $p = q$. The values of the measures D', $|R|$, and $Mvol$ for the table identified by z are presented as ratios between broken and solid lines of the following colors respectively: red, green, and blue.

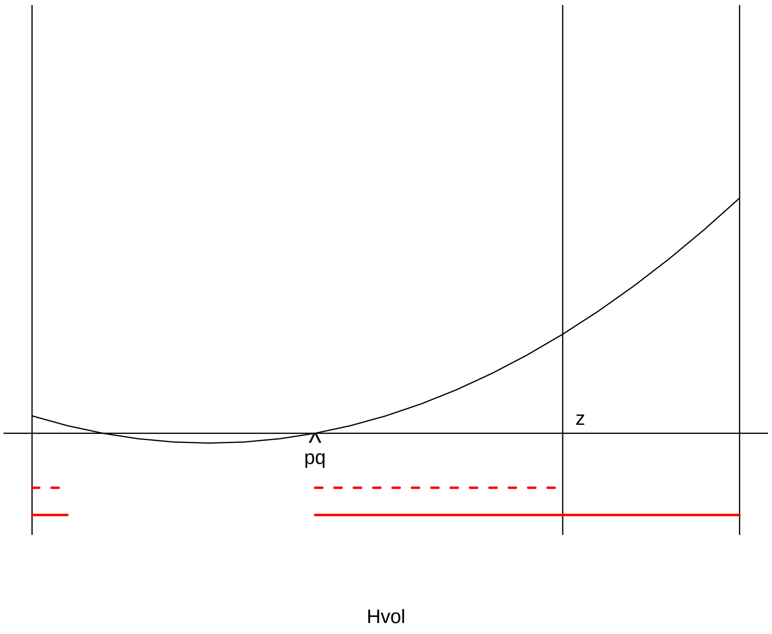

Figure 3: Hvol in 2×2 tables. The value of the x entry of the table in (1) is displayed on the horizontal coordinate. The point z corresponds to a specific table under consideration. The highlighted point pq corresponds to linkage equilibrium (independence). The values of the measure $Hvol$ for the table identified by z is presented as the ratio between broken and solid red line.
